# 1-Benzoyl-3,3-bis­(2-methyl­prop­yl)thio­urea

**DOI:** 10.1107/S1600536811004557

**Published:** 2011-02-12

**Authors:** N. Selvakumaran, R. Karvembu, Seik Weng Ng, Edward R. T. Tiekink

**Affiliations:** a Department of Chemistry, National Institute of Technology, Tiruchirappalli 620 015, India; b Department of Chemistry, University of Malaya, 50603 Kuala Lumpur, Malaysia

## Abstract

The title compound, C_16_H_24_N_2_OS, is twisted about the central N(H)—C bond with the C—N—C—S torsion angle being 119.6 (3)°. The carbonyl O and thione S atoms are directed to opposite sides of the mol­ecule, a conformation that allows for the formation of a linear supra­molecular chain comprising alternating eight-membered {⋯HNCS}_2_ and 14-membered {⋯HCNCNCO}_2_ synthons.

## Related literature

For the coordinating ability of *N*,*N*-dialkyl-*N*′-benzoyl­thio­ureas; see: Binzet *et al.* (2009 ▶); Gunasekaran *et al.* (2010 ▶); Sacht *et al.* (2000 ▶). For the utility of Cd derivatives to serve as synthetic precursors for CdS nanoparticles, see: Bruce *et al.* (2007 ▶). For their biological activity, see: Arslan *et al.* (2006 ▶). For related structures, see: Gunasekaran *et al.* (2010*a* ▶,*b* ▶).
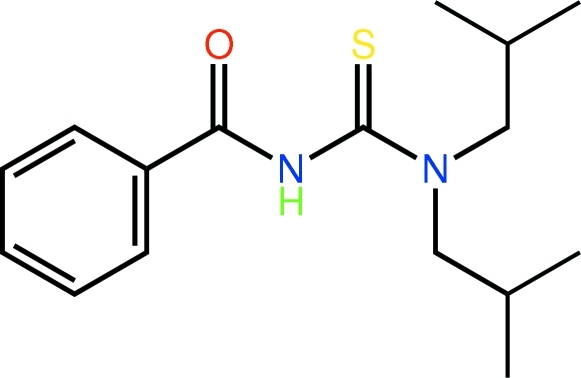


## Experimental

### 

#### Crystal data

C_16_H_24_N_2_OS
*M* *_r_* = 292.43
Triclinic, 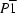

*a* = 8.9331 (10) Å
*b* = 10.1023 (9) Å
*c* = 11.0725 (12) Å
α = 105.776 (9)°
β = 112.734 (10)°
γ = 100.782 (9)°
*V* = 837.47 (19) Å^3^
*Z* = 2
Mo *K*α radiation
μ = 0.19 mm^−1^
*T* = 295 K
0.35 × 0.30 × 0.25 mm

#### Data collection

Agilent Supernova Dual diffractometer with an Atlas detector
Absorption correction: multi-scan (*CrysAlis PRO*; Agilent, 2010 ▶) *T*_min_ = 0.936, *T*_max_ = 0.954
6265 measured reflections
3693 independent reflections
2232 reflections with *I* > 2σ(*I*)
*R*_int_ = 0.025

#### Refinement

*R*[*F* ^2^ > 2σ(*F* ^2^)] = 0.074
*wR*(*F* ^2^) = 0.219
*S* = 1.04
3693 reflections
181 parameters
12 restraints
H-atom parameters constrained
Δρ_max_ = 0.86 e Å^−3^
Δρ_min_ = −0.58 e Å^−3^

### 

Data collection: *CrysAlis PRO* (Agilent, 2010 ▶); cell refinement: *CrysAlis PRO*; data reduction: *CrysAlis PRO*; program(s) used to solve structure: *SHELXS97* (Sheldrick, 2008 ▶); program(s) used to refine structure: *SHELXL97* (Sheldrick, 2008 ▶); molecular graphics: *ORTEP-3* (Farrugia, 1997 ▶) and *DIAMOND* (Brandenburg, 2006 ▶); software used to prepare material for publication: *publCIF* (Westrip, 2010 ▶).

## Supplementary Material

Crystal structure: contains datablocks global, I. DOI: 10.1107/S1600536811004557/ez2230sup1.cif

Structure factors: contains datablocks I. DOI: 10.1107/S1600536811004557/ez2230Isup2.hkl

Additional supplementary materials:  crystallographic information; 3D view; checkCIF report

## Figures and Tables

**Table 1:** Hydrogen-bond geometry (Å, °)

*D*—H⋯*A*	*D*—H	H⋯*A*	*D*⋯*A*	*D*—H⋯*A*
N1—H1⋯S1^i^	0.88	2.74	3.586 (3)	162
C9—H9a⋯O1^ii^	0.97	2.49	3.424 (5)	162

Symmetry codes: (i) 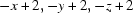; (ii) 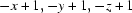.

**Fig. 1.**
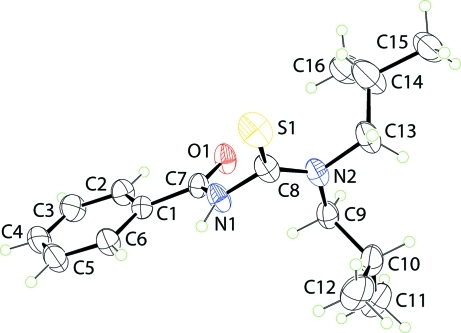
The molecular structure of (I) showing the atom-labelling scheme and displacement ellipsoids at the 35% probability level.

**Fig. 2.**
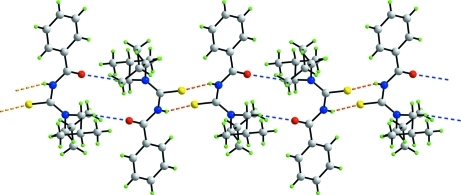
Supramolecular chain in (I) mediated by N–H···S hydrogen bonds and C–H···O contacts, shown as orange and blue dashed lines, respectively.

**Fig. 3.**
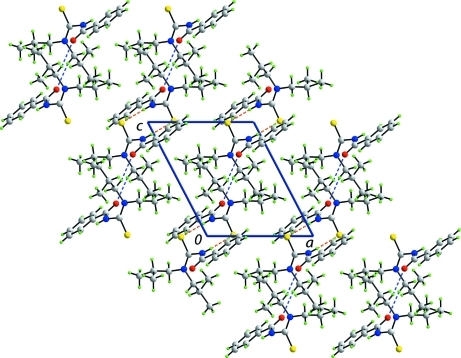
View in projection down the b axis of the unit cell contents of (I). The N–H···S hydrogen bonds and C–H···O contacts are shown as orange and blue dashed lines, respectively.

## supplementary crystallographic information

### Comment

*N*,*N*-Dialkyl-*N*'-benzoylthioureas are versatile ligands which can coordinate to transition metal centres either as neutral species or in an anionic form. The complexation capacity of thiourea derivatives has been reported in several studies (Binzet *et al.*, 2009; Gunasekaran *et al.*, 2010). Chiral and achiral platinum(II) complexes of these ligands have been used as chemotherapeutic agents (Sacht *et al.*, 2000) while cadmium(II) complexes of *N*,*N*-diethyl-*N*'-benzoylthiourea have been used as single-source precursors for the preparation of CdS nanoparticles (Bruce *et al.*, 2007). In addition, thioureas have been shown to possess anti-tubercular, anti-helmintic, anti-bacterial, insecticidal and rodenticidal properties (Arslan *et al.*, 2006). In continuation of structural studies of these derivatives (Gunasekaran *et al.*, 2010*a*; Gunasekaran *et al.*, 2010*b*), the title compound, (I), was investigated.

In (I), Fig. 1, the molecule exhibits a significant twist about the central N(H)–C bond as seen in the value of the C7–N1–C8–S1 torsion angle of 119.6 (3) °. This arrangement causes the carbonyl-O and thione-S atoms to lie on opposite sides of the molecule. Similarly, the carbonyl and N–H groups are directed away from each other. This conformation allows for the formation of N–H···S hydrogen bonds, Table 1, *via* an eight-membered {···HNCS}_2_ ring which has the shape of an elongated chair. These are connected into supramolecular chains along [1 1 1] *via* C–H···O contacts that close 14-membered {···HCNCNCO}_2_ synthons, also adopting the shape of an elongated chair, Table 1 and Fig. 2. Chains are arranged into layers *via* weak π···π interactions [*Cg*(C1–C6)···*Cg*(C1–C6)^i^ = 3.806 (3) Å for *i*: 2 - *x*, 1 - *y*, 2 - *z*] and these stack as shown in Fig. 3.

### Experimental

A solution of benzoyl chloride (0.7029 g, 5 mmol) in acetone (50 ml) was added drop wise to a suspension of potassium thiocyanate (0.4859 g, 5 mmol) in anhydrous acetone (50 ml). The reaction mixture was heated under reflux for 45 min. and then cooled to room temperature. A solution of diisobutyl amine (0.6462 g, 5 mmol) in acetone (30 ml) was added and the resulting mixture was stirred for 2 h. Hydrochloric acid (0.1 N, 300 ml) was added and the resulting white solid was filtered, washed with water and dried *in vacuo*. Single crystals for X-ray diffraction were grown at room temperature from an acetonitrile solution of the compound. Yield 72%; *M*.Pt. 415 K. FT—IR (KBr) ν(N—H) 3268, ν(C═O) 1688, ν(C=S) 1264 cm^-1^.

### Refinement

The H-atoms were placed in calculated positions (N—H = 0.88; C—H 0.93 to 0.98 Å) and were included in the refinement in the riding model approximation, with *U*_iso_(H) set to 1.2 to 1.5*U*_equiv_(N, C).

### Crystal data

**Table:**

C_16_H_24_N_2_OS	*Z* = 2
*M**_r_* = 292.43	*F*(000) = 316
Triclinic, *P*1	*D*_x_ = 1.160 Mg m^−^^3^
Hall symbol: -P 1	Mo *K*α radiation, λ = 0.71073 Å
*a* = 8.9331 (10) Å	Cell parameters from 1845 reflections
*b* = 10.1023 (9) Å	θ = 2.2–29.2°
*c* = 11.0725 (12) Å	µ = 0.19 mm^−^^1^
α = 105.776 (9)°	*T* = 295 K
β = 112.734 (10)°	Block, colourless
γ = 100.782 (9)°	0.35 × 0.30 × 0.25 mm
*V* = 837.47 (19) Å^3^	

### Data collection

**Table:**

Agilent Supernova Dual diffractometer with an Atlas detector	3693 independent reflections
Radiation source: SuperNova (Mo) X-ray Source	2232 reflections with *I* > 2σ(*I*)
Mirror	*R*_int_ = 0.025
Detector resolution: 10.4041 pixels mm^-1^	θ_max_ = 27.5°, θ_min_ = 2.2°
ω scans	*h* = −10→11
Absorption correction: multi-scan (*CrysAlis PRO*; Agilent, 2010)	*k* = −13→12
*T*_min_ = 0.936, *T*_max_ = 0.954	*l* = −14→12
6265 measured reflections	

### Refinement

**Table:**

Refinement on *F*^2^	Primary atom site location: structure-invariant direct methods
Least-squares matrix: full	Secondary atom site location: difference Fourier map
*R*[*F*^2^ > 2σ(*F*^2^)] = 0.074	Hydrogen site location: inferred from neighbouring sites
*wR*(*F*^2^) = 0.219	H-atom parameters constrained
*S* = 1.04	*w* = 1/[σ^2^(*F*_o_^2^) + (0.078*P*)^2^ + 0.7593*P*] where *P* = (*F*_o_^2^ + 2*F*_c_^2^)/3
3693 reflections	(Δ/σ)_max_ = 0.001
181 parameters	Δρ_max_ = 0.86 e Å^−^^3^
12 restraints	Δρ_min_ = −0.58 e Å^−^^3^

### Special details

**Table:**

Geometry. All e.s.d.'s (except the e.s.d. in the dihedral angle between two l.s. planes) are estimated using the full covariance matrix. The cell e.s.d.'s are taken into account individually in the estimation of e.s.d.'s in distances, angles and torsion angles; correlations between e.s.d.'s in cell parameters are only used when they are defined by crystal symmetry. An approximate (isotropic) treatment of cell e.s.d.'s is used for estimating e.s.d.'s involving l.s. planes.
Refinement. Refinement of *F*^2^ against ALL reflections. The weighted *R*-factor *wR* and goodness of fit *S* are based on *F*^2^, conventional *R*-factors *R* are based on *F*, with *F* set to zero for negative *F*^2^. The threshold expression of *F*^2^ > 2σ(*F*^2^) is used only for calculating *R*-factors(gt) *etc*. and is not relevant to the choice of reflections for refinement. *R*-factors based on *F*^2^ are statistically about twice as large as those based on *F*, and *R*- factors based on ALL data will be even larger.

### Fractional atomic coordinates and isotropic or equivalent isotropic displacement parameters (Å^2^)

**Table:**

	*x*	*y*	*z*	*U*_iso_*/*U*_eq_	
S1	0.74828 (14)	0.92803 (11)	0.99096 (12)	0.0711 (4)	
O1	0.6998 (3)	0.5295 (2)	0.7153 (3)	0.0585 (7)	
N1	0.8690 (3)	0.7638 (3)	0.8494 (3)	0.0512 (7)	
H1	0.9753	0.8236	0.8909	0.061*	
N2	0.6172 (3)	0.7981 (3)	0.7098 (3)	0.0515 (7)	
C1	0.9961 (4)	0.5699 (3)	0.8524 (3)	0.0431 (7)	
C2	0.9697 (5)	0.4222 (4)	0.8112 (4)	0.0535 (9)	
H2	0.8597	0.3566	0.7487	0.064*	
C3	1.1045 (5)	0.3706 (4)	0.8615 (5)	0.0653 (11)	
H3	1.0847	0.2708	0.8335	0.078*	
C4	1.2664 (5)	0.4656 (5)	0.9522 (5)	0.0669 (11)	
H4	1.3568	0.4306	0.9866	0.080*	
C5	1.2961 (5)	0.6133 (5)	0.9929 (5)	0.0661 (11)	
H5	1.4069	0.6780	1.0542	0.079*	
C6	1.1618 (4)	0.6657 (4)	0.9431 (4)	0.0538 (9)	
H6	1.1826	0.7656	0.9705	0.065*	
C7	0.8421 (4)	0.6165 (3)	0.7986 (3)	0.0426 (7)	
C8	0.7372 (4)	0.8250 (3)	0.8392 (4)	0.0513 (9)	
C9	0.6351 (4)	0.7432 (3)	0.5811 (4)	0.0526 (9)	
H9A	0.5352	0.6588	0.5122	0.063*	
H9B	0.7353	0.7118	0.6035	0.063*	
C10	0.6535 (5)	0.8564 (4)	0.5150 (4)	0.0654 (11)	
H10	0.5463	0.8792	0.4831	0.079*	
C11	0.6790 (7)	0.7906 (5)	0.3856 (4)	0.0892 (15)	
H11A	0.5849	0.7025	0.3204	0.134*	
H11B	0.7847	0.7691	0.4149	0.134*	
H11C	0.6835	0.8587	0.3403	0.134*	
C12	0.7998 (6)	0.9970 (4)	0.6237 (5)	0.0877 (15)	
H12A	0.8091	1.0661	0.5801	0.132*	
H12B	0.9057	0.9765	0.6583	0.132*	
H12C	0.7765	1.0367	0.7012	0.132*	
C13	0.4659 (4)	0.8426 (4)	0.6909 (5)	0.0662 (11)	
H13A	0.4206	0.8536	0.6005	0.079*	
H13B	0.5025	0.9377	0.7640	0.079*	
C14	0.3240 (5)	0.7441 (4)	0.6949 (7)	0.106 (2)	
H14	0.3640	0.7625	0.7957	0.127*	
C15	0.1645 (5)	0.7914 (5)	0.6518 (6)	0.0888 (15)	
H15A	0.1979	0.8950	0.6987	0.133*	
H15B	0.0863	0.7435	0.6785	0.133*	
H15C	0.1094	0.7655	0.5512	0.133*	
C16	0.2816 (6)	0.5830 (4)	0.6261 (6)	0.0816 (13)	
H16A	0.3853	0.5586	0.6554	0.122*	
H16B	0.2264	0.5542	0.5251	0.122*	
H16C	0.2058	0.5330	0.6536	0.122*	

### Atomic displacement parameters (Å^2^)

**Table:**

	*U*^11^	*U*^22^	*U*^33^	*U*^12^	*U*^13^	*U*^23^
S1	0.0676 (7)	0.0507 (6)	0.0765 (7)	0.0191 (5)	0.0294 (6)	0.0036 (5)
O1	0.0434 (13)	0.0383 (13)	0.0653 (16)	0.0071 (11)	0.0031 (12)	0.0157 (12)
N1	0.0344 (14)	0.0355 (15)	0.0622 (18)	0.0091 (12)	0.0100 (13)	0.0078 (13)
N2	0.0364 (14)	0.0400 (15)	0.0659 (19)	0.0137 (12)	0.0144 (14)	0.0156 (14)
C1	0.0428 (17)	0.0444 (18)	0.0423 (17)	0.0167 (15)	0.0181 (14)	0.0176 (14)
C2	0.054 (2)	0.0427 (19)	0.059 (2)	0.0190 (16)	0.0215 (17)	0.0169 (17)
C3	0.069 (3)	0.054 (2)	0.088 (3)	0.034 (2)	0.040 (2)	0.034 (2)
C4	0.055 (2)	0.077 (3)	0.089 (3)	0.039 (2)	0.035 (2)	0.046 (3)
C5	0.0419 (19)	0.071 (3)	0.077 (3)	0.0192 (19)	0.0186 (18)	0.029 (2)
C6	0.0451 (19)	0.050 (2)	0.060 (2)	0.0171 (16)	0.0180 (16)	0.0193 (17)
C7	0.0417 (17)	0.0384 (17)	0.0383 (16)	0.0125 (14)	0.0111 (14)	0.0128 (14)
C8	0.0403 (17)	0.0325 (17)	0.067 (2)	0.0098 (14)	0.0173 (16)	0.0118 (16)
C9	0.0438 (18)	0.0412 (19)	0.060 (2)	0.0136 (15)	0.0125 (16)	0.0183 (17)
C10	0.055 (2)	0.052 (2)	0.075 (3)	0.0160 (18)	0.0104 (19)	0.032 (2)
C11	0.113 (4)	0.077 (3)	0.071 (3)	0.025 (3)	0.033 (3)	0.038 (3)
C12	0.092 (3)	0.052 (3)	0.089 (3)	0.000 (2)	0.021 (3)	0.030 (2)
C13	0.046 (2)	0.057 (2)	0.089 (3)	0.0247 (18)	0.022 (2)	0.027 (2)
C14	0.065 (3)	0.072 (3)	0.203 (7)	0.033 (3)	0.070 (4)	0.065 (4)
C15	0.060 (3)	0.097 (4)	0.139 (5)	0.039 (3)	0.057 (3)	0.060 (4)
C16	0.071 (3)	0.057 (3)	0.113 (4)	0.012 (2)	0.047 (3)	0.027 (3)

### Geometric parameters (Å, °)

**Table:**

S1—C8	1.673 (4)	C9—H9B	0.9700
O1—C7	1.214 (4)	C10—C12	1.528 (4)
N1—C7	1.377 (4)	C10—C11	1.529 (4)
N1—C8	1.410 (4)	C10—H10	0.9800
N1—H1	0.8800	C11—H11A	0.9600
N2—C8	1.330 (4)	C11—H11B	0.9600
N2—C13	1.461 (4)	C11—H11C	0.9600
N2—C9	1.464 (5)	C12—H12A	0.9600
C1—C2	1.380 (5)	C12—H12B	0.9600
C1—C6	1.386 (5)	C12—H12C	0.9600
C1—C7	1.493 (4)	C13—C14	1.482 (4)
C2—C3	1.381 (5)	C13—H13A	0.9700
C2—H2	0.9300	C13—H13B	0.9700
C3—C4	1.362 (6)	C14—C16	1.498 (4)
C3—H3	0.9300	C14—C15	1.530 (4)
C4—C5	1.376 (6)	C14—H14	0.9800
C4—H4	0.9300	C15—H15A	0.9600
C5—C6	1.382 (5)	C15—H15B	0.9600
C5—H5	0.9300	C15—H15C	0.9600
C6—H6	0.9300	C16—H16A	0.9600
C9—C10	1.533 (4)	C16—H16B	0.9600
C9—H9A	0.9700	C16—H16C	0.9600
			
C7—N1—C8	124.2 (3)	C12—C10—H10	108.2
C7—N1—H1	117.9	C11—C10—H10	108.2
C8—N1—H1	117.9	C9—C10—H10	108.2
C8—N2—C13	120.0 (3)	C10—C11—H11A	109.5
C8—N2—C9	124.2 (3)	C10—C11—H11B	109.5
C13—N2—C9	115.2 (3)	H11A—C11—H11B	109.5
C2—C1—C6	118.6 (3)	C10—C11—H11C	109.5
C2—C1—C7	117.4 (3)	H11A—C11—H11C	109.5
C6—C1—C7	124.0 (3)	H11B—C11—H11C	109.5
C1—C2—C3	120.9 (4)	C10—C12—H12A	109.5
C1—C2—H2	119.6	C10—C12—H12B	109.5
C3—C2—H2	119.6	H12A—C12—H12B	109.5
C4—C3—C2	120.1 (4)	C10—C12—H12C	109.5
C4—C3—H3	119.9	H12A—C12—H12C	109.5
C2—C3—H3	119.9	H12B—C12—H12C	109.5
C3—C4—C5	120.0 (3)	N2—C13—C14	116.6 (3)
C3—C4—H4	120.0	N2—C13—H13A	108.1
C5—C4—H4	120.0	C14—C13—H13A	108.1
C4—C5—C6	120.2 (4)	N2—C13—H13B	108.1
C4—C5—H5	119.9	C14—C13—H13B	108.1
C6—C5—H5	119.9	H13A—C13—H13B	107.3
C5—C6—C1	120.2 (3)	C13—C14—C16	118.4 (4)
C5—C6—H6	119.9	C13—C14—C15	111.0 (3)
C1—C6—H6	119.9	C16—C14—C15	112.5 (4)
O1—C7—N1	121.4 (3)	C13—C14—H14	104.5
O1—C7—C1	122.1 (3)	C16—C14—H14	104.5
N1—C7—C1	116.6 (3)	C15—C14—H14	104.5
N2—C8—N1	117.1 (3)	C14—C15—H15A	109.5
N2—C8—S1	125.9 (3)	C14—C15—H15B	109.5
N1—C8—S1	117.1 (3)	H15A—C15—H15B	109.5
N2—C9—C10	113.3 (3)	C14—C15—H15C	109.5
N2—C9—H9A	108.9	H15A—C15—H15C	109.5
C10—C9—H9A	108.9	H15B—C15—H15C	109.5
N2—C9—H9B	108.9	C14—C16—H16A	109.5
C10—C9—H9B	108.9	C14—C16—H16B	109.5
H9A—C9—H9B	107.7	H16A—C16—H16B	109.5
C12—C10—C11	112.2 (3)	C14—C16—H16C	109.5
C12—C10—C9	110.9 (3)	H16A—C16—H16C	109.5
C11—C10—C9	109.1 (3)	H16B—C16—H16C	109.5
			
C6—C1—C2—C3	1.4 (5)	C13—N2—C8—N1	172.5 (3)
C7—C1—C2—C3	−177.1 (3)	C9—N2—C8—N1	−16.3 (5)
C1—C2—C3—C4	−0.5 (6)	C13—N2—C8—S1	−9.0 (5)
C2—C3—C4—C5	−0.5 (6)	C9—N2—C8—S1	162.2 (3)
C3—C4—C5—C6	0.6 (7)	C7—N1—C8—N2	−61.8 (5)
C4—C5—C6—C1	0.4 (6)	C7—N1—C8—S1	119.6 (3)
C2—C1—C6—C5	−1.4 (5)	C8—N2—C9—C10	−109.9 (4)
C7—C1—C6—C5	177.1 (3)	C13—N2—C9—C10	61.7 (4)
C8—N1—C7—O1	15.7 (5)	N2—C9—C10—C12	53.6 (4)
C8—N1—C7—C1	−163.6 (3)	N2—C9—C10—C11	177.6 (3)
C2—C1—C7—O1	−4.2 (5)	C8—N2—C13—C14	−82.1 (5)
C6—C1—C7—O1	177.4 (3)	C9—N2—C13—C14	105.9 (4)
C2—C1—C7—N1	175.2 (3)	N2—C13—C14—C16	−39.2 (7)
C6—C1—C7—N1	−3.3 (5)	N2—C13—C14—C15	−171.4 (4)

### Hydrogen-bond geometry (Å, °)

**Table:**

*D*—H···*A*	*D*—H	H···*A*	*D*···*A*	*D*—H···*A*
N1—H1···S1^i^	0.88	2.74	3.586 (3)	162
C9—H9a···O1^ii^	0.97	2.49	3.424 (5)	162

Symmetry codes: (i) −*x*+2, −*y*+2, −*z*+2; (ii) −*x*+1, −*y*+1, −*z*+1.
